# Author Correction: Decoding accelerometry for classification and prediction of critically ill patients with severe brain injury

**DOI:** 10.1038/s41598-022-05237-4

**Published:** 2022-01-13

**Authors:** Shubhayu Bhattacharyay, John Rattray, Matthew Wang, Peter H. Dziedzic, Eusebia Calvillo, Han B. Kim, Eshan Joshi, Pawel Kudela, Ralph Etienne‑Cummings, Robert D. Stevens

**Affiliations:** 1 Laboratory of Computational Intensive Care Medicine, Johns Hopkins University, Baltimore, MD USA; 2 Department of Clinical Neurosciences, University of Cambridge, Cambridge, UK; 3 Department of Biomedical Engineering, Johns Hopkins University, Baltimore, MD USA; 4 Department of Applied Mathematics and Statistics, Johns Hopkins University, Baltimore, MD USA; 5 Department of Electrical and Computer Engineering, Johns Hopkins University, Baltimore, MD USA; 6 Department of Neurology, Johns Hopkins University, Baltimore, MD USA; 7 Department of Anesthesiology and Critical Care Medicine, Johns Hopkins University, Baltimore, MD USA; 8 Department of Neurosurgery, Johns Hopkins University, Baltimore, MD USA

Correction to: *Scientific Reports* 10.1038/s41598-021-02974-w, published online 08 December 2021

The original version of this Article contained errors in the Affiliations.

Peter H. Dziedzic and Pawel Kudela were incorrectly affiliated with ‘Department of Anesthesiology and Critical Care Medicine, Johns Hopkins University, Baltimore, MD, USA’.

Eusebia Calvillo and Han B. Kim were incorrectly affiliated with ‘Department of Neurosurgery, Johns Hopkins University, Baltimore, MD, USA’.

Pawel Kudela was incorrectly affiliated with ‘Department of Neurology, Johns Hopkins University, Baltimore, MD, USA’.

The correct affiliations for Peter H. Dziedzic are listed below.

Laboratory of Computational Intensive Care Medicine, Johns Hopkins University, Baltimore, MD, USA.

Department of Neurology, Johns Hopkins University, Baltimore, MD, USA.

The correct affiliation for Pawel Kudela is listed below.

Department of Neurosurgery, Johns Hopkins University, Baltimore, MD, USA.

The correct affiliation for Eusebia Calvillo is listed below.

Department of Neurology, Johns Hopkins University, Baltimore, MD, USA.

The correct affiliations for Han B. Kim are listed below.

Laboratory of Computational Intensive Care Medicine, Johns Hopkins University, Baltimore, MD, USA.

Department of Anesthesiology and Critical Care Medicine, Johns Hopkins University, Baltimore, MD, USA.

As a result, the Affiliations have been renumbered.

The original Article has been corrected.

